# GlyNAC (Glycine and N-Acetylcysteine) Supplementation in Old Mice Improves Brain Glutathione Deficiency, Oxidative Stress, Glucose Uptake, Mitochondrial Dysfunction, Genomic Damage, Inflammation and Neurotrophic Factors to Reverse Age-Associated Cognitive Decline: Implications for Improving Brain Health in Aging

**DOI:** 10.3390/antiox12051042

**Published:** 2023-05-04

**Authors:** Premranjan Kumar, Ob W. Osahon, Rajagopal V. Sekhar

**Affiliations:** Translational Metabolism Unit, Section of Endocrinology, Diabetes and Metabolism, Department of Medicine, Baylor College of Medicine, Houston, TX 77030, USA

**Keywords:** aging, cognitive improvement, brain health, GlyNAC supplementation

## Abstract

Cognitive decline frequently occurs with increasing age, but mechanisms contributing to age-associated cognitive decline (ACD) are not well understood and solutions are lacking. Understanding and reversing mechanisms contributing to ACD are important because increased age is identified as the single most important risk factor for dementia. We reported earlier that ACD in older humans is associated with glutathione (GSH) deficiency, oxidative stress (OxS), mitochondrial dysfunction, glucose dysmetabolism and inflammation, and that supplementing GlyNAC (glycine and N-acetylcysteine) improved these defects. To test whether these defects occur in the brain in association with ACD, and could be improved/reversed with GlyNAC supplementation, we studied young (20-week) and old (90-week) C57BL/6J mice. Old mice received either regular or GlyNAC supplemented diets for 8 weeks, while young mice received the regular diet. Cognition and brain outcomes (GSH, OxS, mitochondrial energetics, autophagy/mitophagy, glucose transporters, inflammation, genomic damage and neurotrophic factors) were measured. Compared to young mice, the old-control mice had significant cognitive impairment and multiple brain defects. GlyNAC supplementation improved/corrected the brain defects and reversed ACD. This study finds that naturally-occurring ACD is associated with multiple abnormalities in the brain, and provides proof-of-concept that GlyNAC supplementation corrects these defects and improves cognitive function in aging.

## 1. Introduction

The biggest risk factor for developing cognitive decline is increased age [1,2,3,4]. Age-associated cognitive decline (ACD) is largely subclinical and is often experienced as declines in memory, problem-solving, processing speed and conceptual reasoning [5,6], and is associated with structural changes in the brain [7]. While research has followed a path of determined pursuit investigating the mechanistic underpinnings of clinically diagnosed cognitive impairment associated with Alzheimer’s disease (AD), few studies have investigated ACD as a potential initiating step. There is a compelling urgency to investigate ACD because the population of older adults is rapidly expanding and expected to exceed 2.1 billion by 2050 [8], and this is certain to be accompanied by a parallel increase in the incidence of ACD and AD. Therefore, focusing on mechanisms contributing to cognitive decline in aging and identifying solutions to improve or reverse ACD will (a) have benefits for improving cognitive health in older adults, and (b) could identify potentially viable mechanistic clues for understanding and combating AD and dementia, especially because both aging and AD share several common associations. For example, aging is associated with deficiency of the antioxidant glutathione (GSH) [9,10,11,12,13,14,15,16,17,18,19], elevated oxidative stress (OxS) [9,10,11,12,13,14], impaired mitochondrial function and inflammation [11,13,14,20,21,22,23,24], and these defects also occur in AD [25,26,27,28,29,30,31,32,33,34,35,36,37,38,39,40,41]. GSH has been linked to neuropsychological function [42], suggesting that GSH deficiency in the brain could contribute to neuro-cognitive decline. Glucose is the primary fuel for brain mitochondria to generate energy. Brain glucose hypometabolism is reported both in older adults and in AD [43,44,45,46,47,48,49,50,51,52,53,54]. Abnormal brain glucose metabolism is identified as a potential risk factor for ACD [55] and for conversion to AD [56]. Furthermore, aging and AD are both associated with declines in glucose transporters critical for glucose entry into the brain, astrocytes and neurons [56,57,58,59,60,61,62,63]. Despite awareness of these overlapping abnormalities in aging and AD, little is known about whether these brain defects in aging can be reversed in an effort to improve cognitive function and promote brain health.

In prior translational studies in aged mice and older humans, we reported the presence of elevated GSH deficiency, OxS, mitochondrial dysfunction (with defective expression of mitochondrial complexes, mitochondrial biogenesis (PGC1α) and mitophagy (PINK1)), inflammation and genomic damage in the heart, liver, kidneys, muscle and blood [14,64], and that these defects can be improved/reversed by supplementing GlyNAC (combination of GSH precursor amino-acids glycine and cysteine (provided as N-acetylcysteine, NAC)). Furthermore, we found and reported that GlyNAC supplementation in older adults improved these defects measured at the whole-body level, and also improved cognition [13]. These data raise the question of whether GlyNAC supplementation could improve defects directly in the aging brain and thereby improve cognitive function. Since obtaining brain biopsies from live humans to study defects directly in the brain is not possible, we opted to study old mice as this permits the investigation of brain defects associated with naturally-occurring ACD. Because we previously reported that old C57BL/6J mice and older adults share many age-associated abnormalities [14,22,64], these mice were an ideal to conduct our investigation of cognitive decline and brain abnormalities in aging. We hypothesized that old C57BL/6J mice would have the following: (a) naturally-occurring ACD; (b) specific abnormalities in the brain: GSH deficiency, elevated OxS, deficient availability of glucose transporters responsible for glucose uptake into the brain, neurons and astrocytes, abnormal mitochondrial biogenesis and energetics, impaired mitophagy and autophagy, increased inflammation and elevated genomic damage, and deficient neurotrophic factors; (c) improvement of these mechanistic defects after receiving GlyNAC; (d) improvement in cognition after receiving GlyNAC. We tested our hypotheses in a study of young and old C57BL/6J mice, and provide the study details, results, and discussion of study findings in this manuscript.

## 2. Materials and Methods

### 2.1. Mouse Studies

Rodent study protocols were approved by the Institutional Animal Care and Use Committee (IACUC) at Baylor College of Medicine and adhered to the criteria outlined in the ‘Guide of the Care and Use of Laboratory Animals’.

### 2.2. Study Details

**Figure 1:**
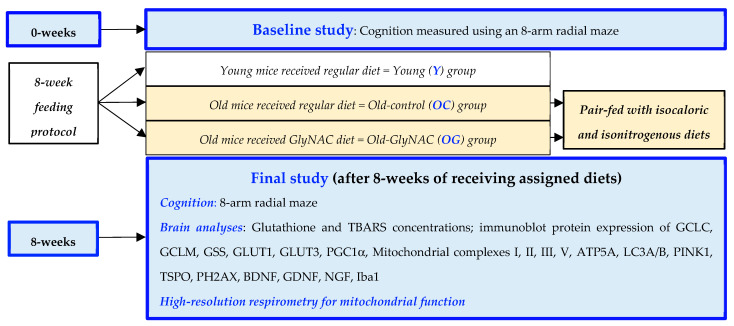
Study experimental details. GCLC and GCLM = catalytic and modifier subunits of glutamate cysteine ligase enzyme; GSS = glutathione synthetase enzyme; GLUT1 and GLUT3 = glucose transporters 1 and 3; PGC1α = peroxisome proliferator-activated receptor-gamma coactivator 1-alpha; ATP5A = mitochondrial adenosine triphosphate synthase F1 subunit alpha; LC3A/B = Light chain 3 microtubule-associated proteins 1A and 1B; PINK1 = PTEN-induced kinase; TSPO = translocator protein; PH2AX = phosphor H2A histone family member X; BDNF = brain-derived neurotrophic factor; GDNF = glial-derived neurotrophic factor; NGF = nerve growth factor.

To conduct the study, 24 male C57BL/6J mice were purchased (The Jackson Laboratory, Bar Harbor, ME, USA [65]), and housed in a dedicated rodent satellite facility with temperature and light control and received daily health monitoring. One group of mice served as young controls (Y, n = 8) and underwent measure of cognitive function (described in Section 2.3.1) before and after receiving a regular diet ad libitum (protein 25.6%, 3.0 kcal/g feed; Harlan Teklad, Indianapolis, IN) for 8 weeks from the age of 20–28 weeks. In the satellite facility, 16 mice were aged until they reached 90 weeks of age when they underwent cognitive testing. The old mice were divided into two groups (8 mice each) matched for cognitive performance. One group of old mice served as old controls (OC, n = 8) for the other group of old mice which were assigned to receive a GlyNAC-supplemented diet (OG, n = 8). The OC mice continued the regular diet (protein 25.6%, 3.0 kcal/g feed; Harlan Teklad, Indianapolis, IN) for 8 weeks. The OG mice were pair-fed to the OC mice with the GlyNAC-supplemented diet (protein 23.5%, 3.0 kcal/g feed, N-acetylcysteine (NAC) 1.6 mg/g feed and glycine 1.6 mg/g feed; Harlan Teklad, Indianapolis, IN) for 8 weeks, keeping the GlyNAC supplemented diet isocaloric and isonitrogenous to the regular diet. These doses of supplemental glycine and NAC in the GlyNAC diet were chosen to approximate doses of GlyNAC supplementation used in human clinical trials in older adults [12,13,64]. After completing 8 weeks on their respective diets, mice underwent cognitive studies again and were then euthanized with immediate collection of the brain for measuring outcomes. Figure 1 provides the experimental details of the study.

### 2.3. Study Outcome Measures

#### 2.3.1. Cognitive Measurement

Cognition was tested using the 8-arm radial maze (8ARM), a commonly and widely used behavioral test in rodents to measure spatial working memory and reference memory [66,67,68,69]. The 8ARM consists of a central chamber/compartment from where 8 arms extend. The arms are equally spaced from one another and are of equal length. One end of an arm contains a food reward that cannot be seen from the central chamber. Habituation is done prior to the test where the mouse is placed in the central chamber and is free to explore the 8ARM and find the reward. During the actual test, a mouse is again placed in the central chamber and allowed to freely explore the arms of the maze in search of the food reward. While exploring the maze, the mouse must remember the arms it visits to ensure avoidance of those arms that do not contain the reward and avoid futile re-entry into empty arms. The maze is designed to ensure that the mouse after visiting any arm must return to the central chamber before it can enter another arm. Therefore, each time the mouse returns to the central chamber it is presented with the same 8-arm options. To find the food reward, the mouse must remember the choices it has made, and avoid making errors. To minimize any distractions, the test is always conducted in a silent room maintained at an ambient temperature of approximately 25 °C with humidity 50 ± 5%, and at the same time of day. The test is scored in two ways, (a) time taken to find the reward; (b) the number of errors made before finding the reward. A correct choice occurs if the mouse enters an unvisited arm followed by food acquisition, and an error is counted if the mouse re-enters a previously visited empty arm.

#### 2.3.2. Brain Glutathione and Oxidative Stress

Brain concentrations of total and reduced GSH (tGSH and rGSH) were measured by ultraperformance liquid chromatography (Waters ACQUITY UPLC System) as reported by us previously [13,14,64]. Oxidized glutathione (GSSG) levels were calculated as the difference between tGSH and rGSH. OxS was measured using a TBARS (thiobarbituric acid reducing substances) assay kit (Cayman Chemicals, Ann Arbor, MI, USA) [70].

#### 2.3.3. Brain High-Resolution Respirometry

Mitochondrial function was estimated by high-resolution respirometry (Oxygraph-2k, Oroboros Instruments, Innsbruck, Austria) using the substrate-uncoupler-inhibitor titration (SUIT) protocol [71]. Briefly, after euthanasia, fresh brain tissue was immediately obtained, homogenized and permeabilized with saponin in cold respiration media (0.5 mM EGTA, 3 mM MgCl2, 60 mM K-lactobionate, 20 mM taurine, 10 mM KH2PO4, 20 mM HEPES, 110 mM sucrose and 0.1% [*w/v*] bovine serum albumin [pH 7.1]). This brain tissue homogenate was added to a 2 mL Oroboros chamber to assess oxygen flux. Pyruvate (10 mM), glutamate (20 mM) and succinate (10 mM) and glycerol-phosphate were added to evaluate ADP-stimulated respiration when NADH and succinate-linked pathways are simultaneously transferring electrons to the Q junction (Oxphos complexes I + II). Subsequently, trifluoromethoxy-carbonylcyanide phenylhydrazone (FCCP) was titrated to achieve maximum flux through the electron transfer system. Then, respiration was inhibited by the sequential addition of rotenone (C-I inhibitor) and antimycin A (Q-CytochromeC inhibitor). The remaining O2 flux after inhibition with antimycin A (O2 flux independent of the electron transfer system) was measured. Finally, respiration was stimulated by providing external electrons via the addition of ascorbate-trimethyphenylenediamine (TMPD), and then complex III was inhibited with sodium azide. Oxygen flux values are expressed relative to tissue weight per second.

#### 2.3.4. Protein Isolation and Immunoblot Analyses in the Brain

Standard techniques were used as reported by us previously [14,64]. Brain tissue from young, old-control and old-GlyNAC mice was used with 40 µg protein lysate per lane. Three sets were done for each blot, with one mouse from each group of Y, OC and OG mice per set. The tissue samples were lysed in 1 x lysis buffer (20 mM Tris-HCL [pH 7.5], 150 mM NaCl, 1 mM Na2EDTA, 1 mM EGTA, 1% Triton X-100, 2.5 mM sodium pyrophosphate, 1 mM b-glycerophosphate, 1 mM Na3VO4, 1 mg/mL leupeptin), supplemented with protease inhibitor 1 mM PMSF. Samples were freeze-thawed followed by centrifugation at 14,000 rpm for 30 min at 4 °C. Protein concentration was measured using Pierce^TM^ BCA Protein Assay Kit (Thermo Scientific, Waltham, MA, USA). Equal amounts of protein lysate were separated by SDS-PAGE and transferred to a polyvinylidene difluoride membrane. The membranes were incubated in 5% *w/v* skimmed milk for 1 h at room temperature followed by primary antibody at 4 °C overnight. The primary antibodies include Glutamate cysteine ligase catalytic and modifier subunits (GCLC, GCLM), Glutathione Synthetase (GSS), Peroxisome proliferator-activated receptor-gamma coactivator (PGC)–1alpha (PGC1α), mitochondrial complexes I-V, mitochondrial adenosine triphosphate (ATP) synthase F1 subunit alpha (ATP5A), Phosphate and tensin homolog (PTEN)-induced kinase 1 (PINK1); Light chain 3 microtubule-associated proteins 1A/1B (LC3A/B); Glucose transporters 1 and 3 (GLUT1, GLUT3), translocator protein (TSPO), phospho-H2A histone family member X (pH2AX), brain-derived neurotrophic factor (BDNF), glial-derived neurotrophic factor (GDNF), nerve growth factor (NGF), and ionized calcium-binding adapter molecule-1 (Iba1). The secondary antibody conjugated with horseradish peroxidase was added for 1 h at room temperature. Membranes were washed with 1X tris buffered saline with Tween^®^20 (TBST) and developed using SuperSignal^TM^ West Dura Extended Duration Substrate (Thermo Scientific, Waltham, MA, USA) on autoradiography film. Optical density was quantified using grayscale measurements in ImageJ 1.51j8 software (NIH, National Institutes of Health) and normalized to the loading control β-actin.

### 2.4. Statistical Analyses

Data are expressed as means ± SE (standard error of the mean). Significance was calculated using 1-way ANOVA and Holm-Šídák multiple-comparisons tests in young-control mice (Y), old-control mice (OC) and old mice supplemented with GlyNAC (OG). Statistical analyses were performed with GraphPad Prism version 8.0 (GraphPad Software, San Diego, CA, USA), and results are considered to be statistically significant at *p* < 0.05.

## 3. Results

### 3.1. Cognitive Function in Young and Old Mice

**Figure 2:**
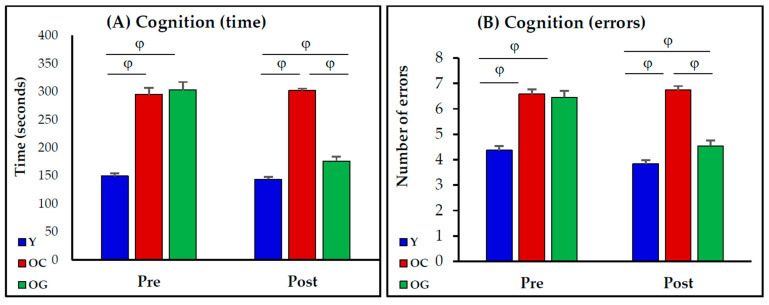
Cognitive function in young mice (Y), old-control mice (OC), and old mice on the GlyNAC diet (OG). Cognition data is shown from before (pre) and after (post) the feeding protocol with 8 mice per group. (**A**) Time taken to complete maze; (**B**) Number of errors during maze completion. φ = *p* < 0.01.

Before beginning supplementation: (a) cognitive performance in old mice was lower than in young mice (old mice took 100% longer (*p* < 0.0001) with 50% more errors (*p* < 0.0001)); (b) there were no differences between the old mice assigned to receive the regular (placebo) diet and the old mice assigned to receive the GlyNAC diet for the time taken (*p* = 0.6), and errors made (*p* = 0.7) in completing the maze. After receiving the assigned diets for 8 weeks, the old mice receiving GlyNAC took less time (42% improvement, *p* < 0.0001) and made less errors (33% improvement, *p* < 0.0001) in completing the maze. However, the cognitive outcomes in the young mice were better than the GlyNAC-supplemented old mice both for time taken (*p* < 0.01) and errors made (*p* < 0.01) in completing the maze. No improvements were seen in old mice receiving the regular diet (Figure 2).

### 3.2. Brain Glutathione

#### 3.2.1. GSH Concentrations in the Brain

**Figure 3:**
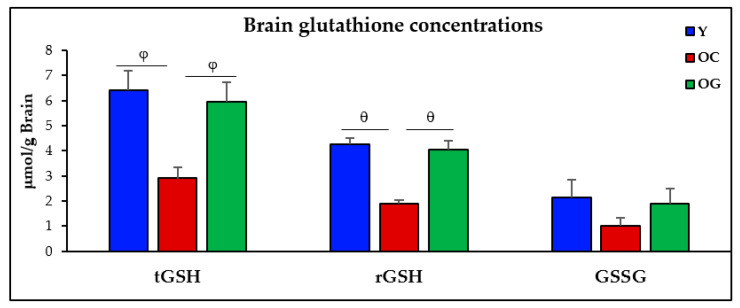
Glutathione concentrations in the brains (n = 8 per group) of young mice (Y), old-control mice on the regular diet (OC) and old mice on the GlyNAC supplemented diet (OG). t-GSH, r-GSH and GSSG = total, reduced and oxidized glutathione concentrations. φ = *p* < 0.01; θ = *p* < 0.001.

The old-control mice had 69% (*p* < 0.001) lower total-GSH concentrations, and 75% (*p* < 0.05) lower reduced-GSH concentrations compared to young mice. Compared to old-control mice, the GlyNAC supplemented old mice had 156% higher total-GSH concentrations (*p* < 0.01) and 204% higher reduced-GSH concentrations (*p* < 0.001), and these results were not statistically different from values in young mice. There were no differences in oxidized glutathione concentrations (GSSG) between the three groups (Figure 3, Table 1). 

#### 3.2.2. GSH Synthesis in the Brain

**Figure 4:**
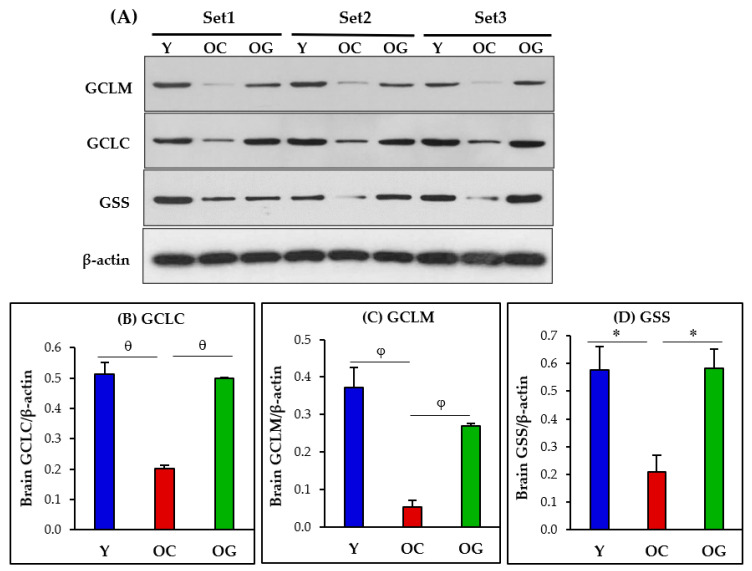
(**A**) Immunoblots for protein expression of GCLC, GCLM and GSS. Each blot represents 3 sets from the brains of young mice (Y), old-control mice (OC) and old mice on GlyNAC supplementation (OG). GCLC and GCLM = Glutamate cysteine ligase, catalytic (GCLC) and modifier (GCLM) subunits; GSS = Glutathione Synthetase. (**B**–**D**) Immunoblot quantification: optical density of protein expression was normalized to the loading control (β-actin). ∗ = *p* < 0.05; φ = *p* < 0.01, θ = *p* < 0.001.

GSH synthesis in the brain was assessed as protein expression of the enzymes of GSH synthesis. These include the catalytic and modifier subunits of glutamate-cysteine ligase (GCLC, GCLM) as the key rate-limiting enzyme of the first step of GSH synthesis, and glutathione synthetase (GSS), the enzyme catalyzing the final step of GSH synthesis. Compared to young mice, old-control mice had significantly lower protein expression of GCLC (*p* < 0.05), GCLM (*p* < 0.05) and GSS (*p* < 0.05). Compared to old-control mice, the GlyNAC-supplemented old mice had significantly higher protein expression of GCLC (*p* < 0.01), GCLM (*p* < 0.05) and GSS (*p* < 0.05) in the brain, and these results were not statistically different from values in young mice (Figure 4).

### 3.3. Oxidative Stress in the Brain

**Figure 5:**
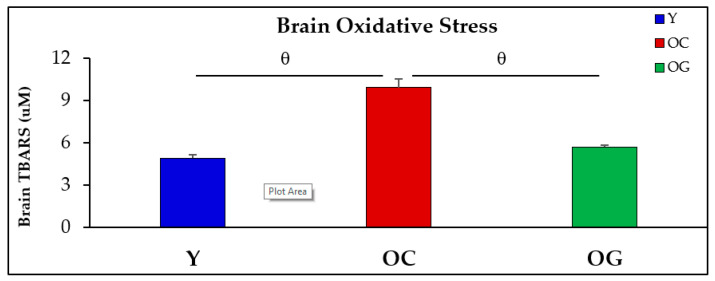
Brain oxidative stress (shown as brain concentrations of TBARS, n = 8 per group) in young mice (Y), old-control mice (OC) and old mice on the GlyNAC-supplemented diet (OG). Results are reported as mean ± SE (standard error). θ = *p* < 0.001.

Oxidative stress was measured in the brains as concentrations of TBARS [69]. Compared to young mice, concentrations of brain TBARS were significantly higher in the brains of old-control mice by 89.7% (4.9 ± 0.2 vs. 9.9 ± 0.6, *p* < 0.001). Compared to the old-control mice, the TBARS concentrations were 42% lower in the brains of old mice supplemented with GlyNAC (9.9 ± 0.6 vs. 5.7 ± 0.2, *p* < 0.001), and these results were not statistically different from values in young mice (Figure 5).

### 3.4. Mitochondrial Function in the Brain

#### 3.4.1. Mitochondrial Protein Immunoblotting

**Figure 6:**
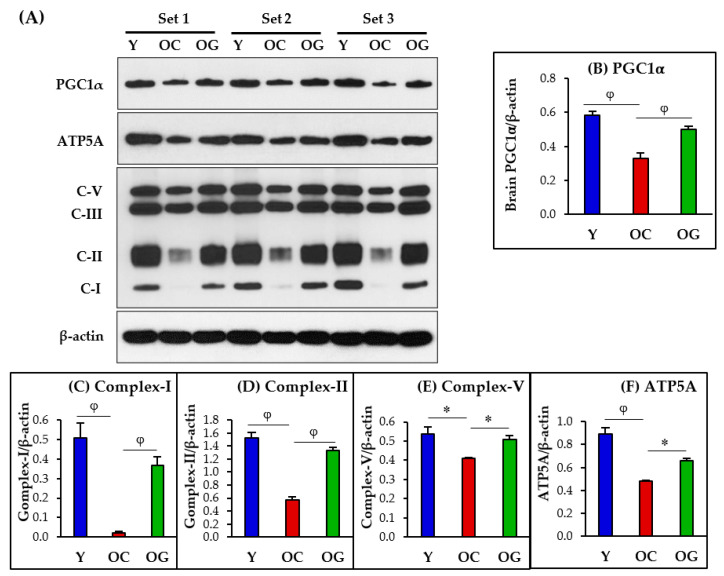
(**A**) Immunoblots for protein expression of PGC1α, mitochondrial complexes and ATP5A. Each blot represents 3 sets from the brains of young mice (Y), old-control mice (OC), and old mice on GlyNAC supplementation (OG). (**B**–**F**) Immunoblot quantification: optical density of protein expression was normalized to the loading control (β-actin). ∗ = *p* < 0.05; φ = *p* < 0.01.

Protein expression of key regulators of mitochondrial biogenesis, mitochondrial energetics and ATP synthesis were significantly lower in the brains of old-control mice compared to young mice (measured as PGC1α, *p* < 0.01; complex-I, *p* < 0.01; complex-II, *p* < 0.01; complex-V, *p* < 0.05; and ATP5A, *p* < 0.001). Compared to old-control mice, GlyNAC supplemented old mice had significantly higher protein expression of PGC1α (*p* < 0.01), mitochondrial complexes I, II and V (*p* < 0.01, *p* < 0.001, *p* < 0.05) and ATP5A (*p* < 0.01), and these values did not differ statistically from young mice (except for ATP5A which remained significantly lower than young mice (*p* < 0.01)). The improvement of complex III protein expression in OG mice did not reach statistical significance (Figure 6).

#### 3.4.2. Mitochondrial Respiration

**Figure 7:**
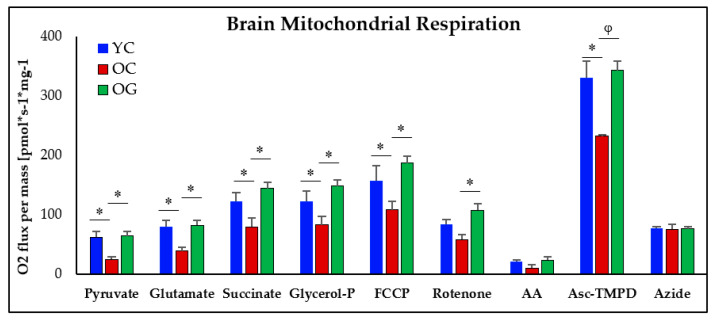
High-resolution respirometry in the brains of young control (Y), old-control (OC) and old mice receiving GlyNAC (OG) with 3 mice studied per group. AA = antimycin-A; Asc-TMPD = Ascorbate-trimethyphenylenediamine; Glycerol-P = glycerol-phosphate. ∗ = *p* < 0.05; φ = *p* < 0.01.

Compared to the brains of young mice, the brains of old-control mice had lower oxygen consumption rate (OCR, Figure 7) after the addition of the fuel substrates pyruvate (*p* < 0.05), glutamate (*p* < 0.05), succinate (*p* < 0.05) and glycerol-phosphate (*p* < 0.05) indicating impairment of mitochondrial complexes I and II. Compared to old-control mice, the brains of old mice receiving GlyNAC had significantly higher OCR after the addition of pyruvate (*p* < 0.05), glutamate (*p* < 0.05), succinate (*p* < 0.05) and glycerol-phosphate (*p* < 0.05) indicating improvement of complexes I and II. The response to the addition of FCCP (to induce uncoupling) also showed an impairment in the brains of aged mice. The OCR response to the addition of FCCP and the complex-I inhibitor Rotenone was significantly higher in the GlyNAC supplemented old mice. After the addition of the inhibitor antimycin-A (inhibits electron flow by inhibiting Q-cytochromeC), ascorbate-TMPD was added to supply electrons: the old-control mice had a lower OCR response than young mice after the addition of ascorbate-TMPD (*p* < 0.05) indicating functional impairment of complex III, but the response in old-mice receiving GlyNAC was significantly higher than old-control mice (*p* < 0.01) indicating improvement of complex-III function. There were no differences between old mice receiving GlyNAC and young mice for these outcomes. After adding sodium azide the responses to all three groups were identical.

### 3.5. Mitophagy and Autophagy in the Brain

**Figure 8:**
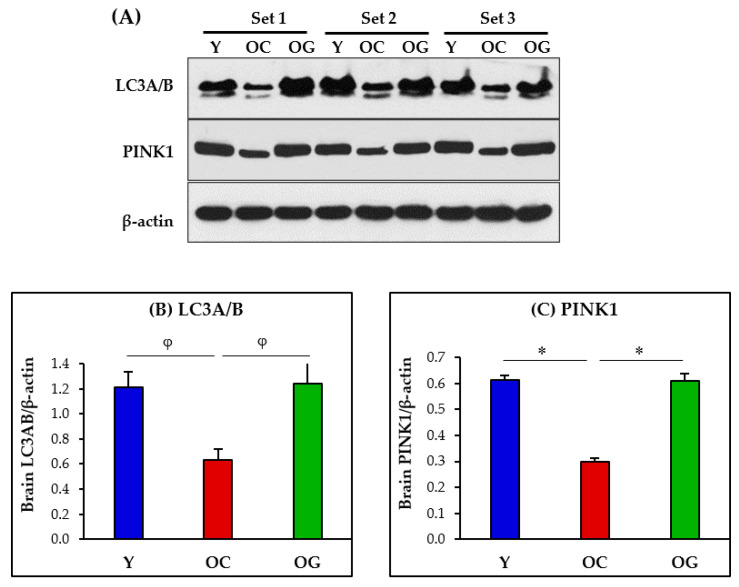
(**A**) Immunoblots for protein expression for LC3A/B and PINK1. Each blot represents 3 sets from the brains of young mice (Y), old-control mice (OC) and old mice on GlyNAC supplementation (OG). PINK1 = PTEN-induced kinase 1; LC3A/B = Light chain 3 microtubule associated proteins 1A/1B. (**B**,**C**) Immunoblot quantification: optical density of protein expression was normalized to the loading control (β-actin). ∗ = *p* < 0.05; φ = *p* < 0.01.

The brains of old-control mice had significantly lower protein expressions of LC3A/B (indicative of LC3-I and LC3-II) (*p* < 0.05) and PINK1 (*p* < 0.001) compared to young mice (Figure 8). After receiving GlyNAC supplementation old mice had significantly higher brain expression of LC3A/B (*p* < 0.05) and PINK1 (*p* < 0.001) compared to old-control mice, and these improvements did not differ from young mice.

### 3.6. Glucose Uptake in the Brain

**Figure 9:**
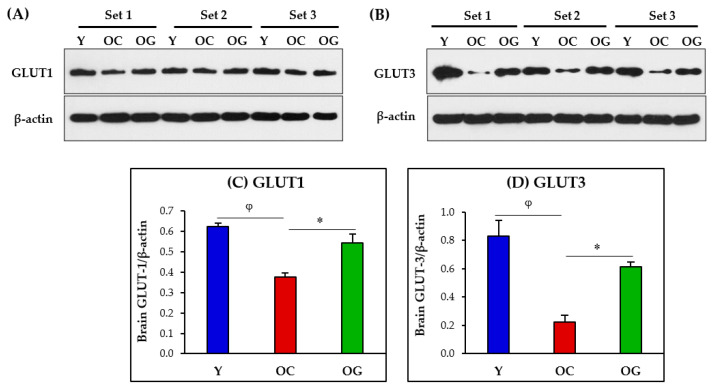
(**A**,**B**) Immunoblots for protein expression of GLUT1 and GLUT3. Each blot represents 3 sets from the brains of young mice (Y), old-control mice (OC), and old mice on GlyNAC supplementation (OG). GLUT1 = Glucose-transporter 1; GLUT3 = Glucose-transporter 3. (**C**,**D**) Immunoblot quantification: optical density of protein expression was normalized to the loading control (β-actin). ∗ = *p* < 0.05; φ = *p* < 0.01.

Glucose uptake into the brain, astrocytes and neurons were assessed as protein expressions of glucose transporters GLUT1 (expressed in the blood-brain barrier and astrocytes) and GLUT3 (expressed in neurons), Figure 9. The brains of old-control mice had significantly lower protein expressions of GLUT1 (*p* < 0.01) and GLUT3 (*p* < 0.01) compared to young mice. Compared to old-control mice, the brains of GlyNAC-supplemented old mice had significantly higher protein expression of GLUT1 (*p* < 0.05) and GLUT3 (*p* < 0.05), and these results were not significantly different from young mice.

### 3.7. Brain Inflammation

**Figure 10:**
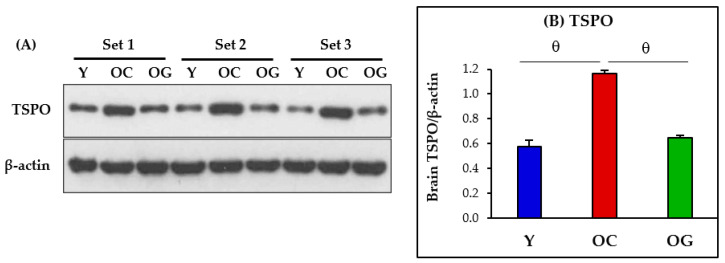
(**A**) Immunoblots for protein expression of TSPO. Each blot represents 3 sets from the brains of young mice (Y), old-control mice (OC, and old mice on GlyNAC supplemented diet (OG). TSPO = Translocator protein, an index of inflammation. (**B**) Immunoblot quantification: optical density of protein expression was normalized to the loading control (β-actin). θ = *p* < 0.0001.

Inflammation in the brain assessed as protein expression of TSPO was significantly higher in the brains of old-control mice compared to young mice (*p* < 0.0001). Compared to old-control mice, TSPO expression was significantly lower in old mice supplemented with GlyNAC (*p* < 0.0001), and these results did not differ from young mice (Figure 10).

### 3.8. Genomic Damage in the Brain

**Figure 11:**
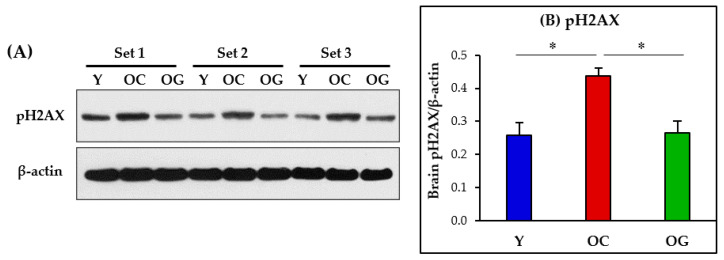
(**A**) Immunoblot of protein expression of pH2AX. The blot represents 3 sets from the brains of young (Y), old-control (OC), and old mice on GlyNAC (OG). (**B**) Immunoblot quantification: optical density of protein expression was normalized to the loading control (β-actin). ∗ = *p* < 0.05.

Genomic damage in the brain was assessed as protein expression of pH2AX (phospho-H2A histone family member X). Compared to young mice, the old-control mice had significantly higher protein expression (*p* < 0.05) of pH2AX in the brain. Compared to old-control mice, GlyNAC supplementation significantly lowered pH2AX protein expression in the brain (*p* < 0.05), and these results did not differ from young mice (Figure 11).

### 3.9. Brain Neurotrophic Factors

**Figure 12:**
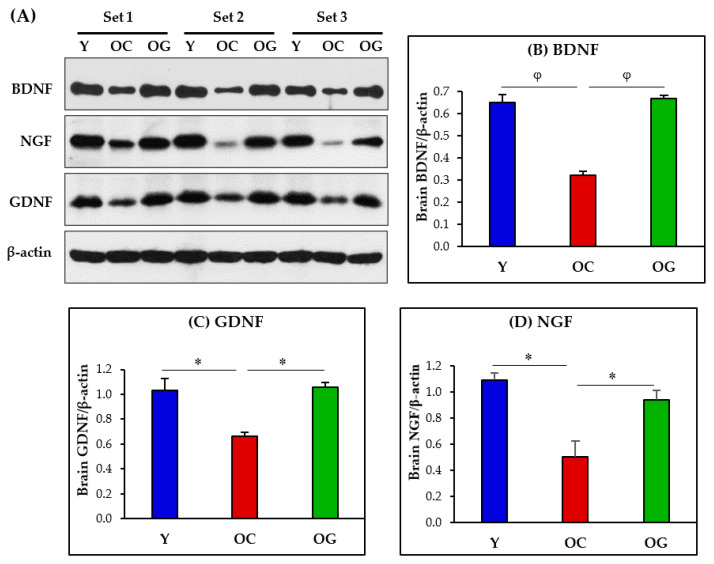
(**A**) Immunoblots for protein expression of BDNF, GDNF and NGF. Each blot represents 3 sets from the brains of young mice (Y), old-control mice (OC) and old mice on GlyNAC supplementation (OG). BDNF = brain-derived neurotrophic factor; GDNF = glial-derived neurotrophic factor; NGF = nerve growth factor. (**B**–**D**) Immunoblot quantification: optical density of protein expression was normalized to the loading control (β-actin). ∗ = *p* < 0.05; φ = *p* < 0.01.

Neurotrophic factors in the brain were assessed as protein expression of BDNF, GDNF and NGF. Compared to young mice, the old-control mice had significantly lower protein expression of BDNF (*p* < 0.01), GDNF (*p* < 0.05) and NGF (*p* < 0.05) in the brain. Compared to the old-control mice, GlyNAC supplemented old mice had significantly higher brain protein expression of BDNF *p* < 0.001), GDNF (*p* < 0.05) and NGF (*p* < 0.05), and these values did not differ from the young mice (Figure 12).

### 3.10. Microglial Activation in the Brain

**Figure 13:**
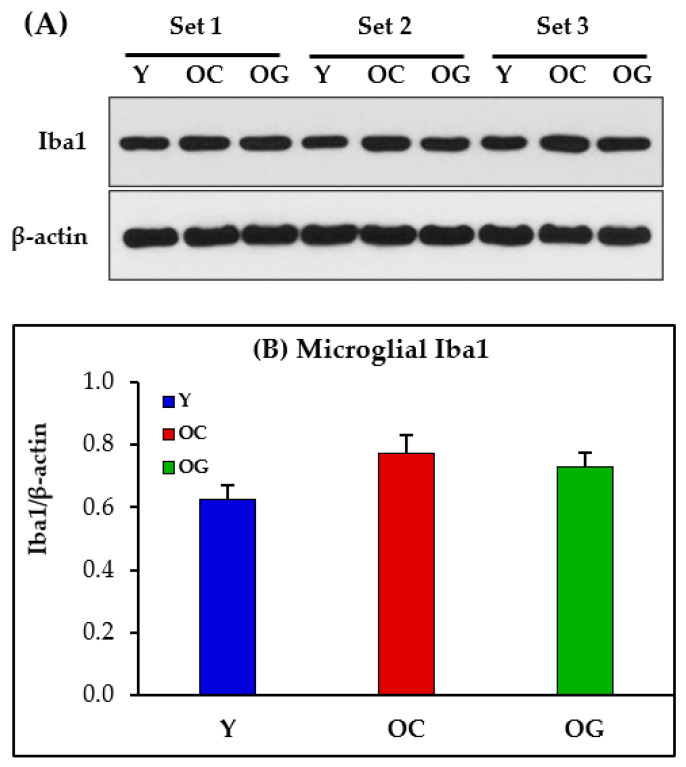
(**A**) Immunoblots for protein expression of brain Iba1. Each blot represents 3 sets from the brains of young mice (Y), old-control mice (OC) and old mice on GlyNAC supplementation (OG). (**B**) Immunoblot quantification: optical density of protein expression was normalized to the loading control (β-actin). Iba1 = Microglial ionized calcium-binding adapter molecule-1. Results were not statistically significant between the three groups.

Microglial activation is postulated as a reason for increased brain inflammation. This study measured the protein expression of Iba1 as an assessment of microglial function (Figure 13) and did not find any statistically significant differences in the protein expression of Iba1 in the brains of old mice compared to young mice.

## 4. Discussion

The key findings of this study are that (1) compared to young mice, old mice have (a) cognitive impairment, (b) brain abnormalities with GSH deficiency, elevated OxS, impaired mitochondrial function, abnormal mitophagy and autophagy, diminished glucose transporters/uptake, elevated inflammation and higher genomic damage; (c) low brain neurotrophic factors (BDNF, GDNF and NGF); and (2) supplementing old mice with GlyNAC improved/reversed these brain defects, and improved cognition.

### 4.1. Rationale for Studying Cognitive Impairment and Associated Brain Defects in Aged Wild-Type Mice

Increased age is recognized as the biggest risk factor for cognitive impairment [1,2,3,4]. Since mice do not naturally develop AD, rodent studies of AD have been primarily conducted in mice which are genetically manipulated via overexpression/knockout of various proteins of interest linked to AD to induce cognitive impairment. Because such studies result in cognitive impairment via external manipulation, they are not representative of naturally-occurring cognitive decline. Evaluation of mechanisms contributing to naturally-occurring cognitive impairment (as seen in aging) could offer valuable insights into the origins of cognitive decline, and also offer an opportunity to test interventions to reverse contributing mechanistic defects toward improving cognition. For these reasons, we conducted this study in aged wild-type C57BL/6J mice to investigate naturally-occurring age-associated cognitive decline (ACD), and also evaluate defects in the aging brain which are associated with ACD. Because an association does not indicate causality, we tested the strength of these associations by also evaluating whether specific supplementation with GlyNAC could improve/reverse brain defects and also improve cognition. This rodent study was guided by the findings in our published open-label trial in older humans where we reported age-associated cognitive decline (detected only on cognitive testing, where participants did not have any prior diagnosis of cognitive impairment) and decreased circulating BDNF in association with GSH deficiency, elevated OxS, mitochondrial dysfunction, increased systemic insulin resistance, inflammation, and decreased brain glucose availability [13]. GlyNAC supplementation for 24 weeks in these older humans improved these defects, and also improved cognition and circulating BDNF levels [13]. Stopping GlyNAC led to a decline/loss of benefits, implying causality with GlyNAC. Because it is not feasible or practical to obtain brain biopsies from live humans, this rodent study permits the study of brain defects in association with ACD, and complements our published human trial by investigating similar defects and the response to GlyNAC supplementation. The study findings and their implications on ACD are discussed below.

### 4.2. GlyNAC Supplementation Improves Age-Associated Cognitive Decline

Cognitive decline and dementia are identified as key contributors to disability in the elderly [72], and increased age is repeatedly identified as the most important risk factor for cognitive decline and dementia [1,2,3,4]. A systematic review of 51 unique trials employing various interventional strategies including dementia medications, antihypertensives, diabetes medications, nonsteroidal anti-inflammatory drugs, aspirin, hormones and lipid-lowering drugs did not find evidence for cognitive protection [73]. The expectation of an increase in the prevalence of cognitive decline and AD due to an anticipated rise in the global aging population [8] has led to calls for a new research roadmap to address dementia [74]. Such a rethink will have to focus on understanding why or how ACD occurs naturally and occurs before the onset of dementia. This approach can facilitate the discovery of effective solutions to combat and reverse cognitive impairment in aging, and thereby prevent the progression to dementia. Our current study in aged mice finds that (a) aged mice develop cognitive decline which is associated with multiple specific defects in the brain, and (b) supplementing GlyNAC successfully reverses multiple brain defects and reverses cognitive decline. These twin benefits provide evidence that GlyNAC supplementation could promote brain health and reverse cognitive decline in aging. However, after GlyNAC supplementation, the improvement in cognitive function remained lower than values in young mice suggesting that the duration of supplementation may have been too short. Future studies should test whether longer durations of GlyNAC supplementation could further improve the cognitive performance of old mice to match young mice. It is also necessary to remember that mice and humans are separate species, and these findings should be confirmed in older humans. In an earlier pilot clinical trial in older adults we found and reported that GlyNAC supplementation improved cognitive function and similar underlying defects, but this trial was limited by a small sample size and lacked a placebo control arm [13]. Larger placebo controlled, double blinded randomized clinical trials in older adults are necessary to definitively confirm these observations.

### 4.3. GlyNAC Supplementation Corrects GSH Deficiency and OxS in the Brain

#### 4.3.1. Glutathione (GSH)

Aging is associated with a deficiency of GSH [9,10,11,12,13,14,15,16,17,18,19], the most abundant intracellular antioxidant. We discovered and reported that GSH deficiency in older humans and aged mice occurs due to diminished synthesis, and can be corrected by supplementing GlyNAC [11,12,13,64]. Interestingly, GSH has been linked to neuropsychological function [42], suggesting that GSH deficiency could contribute to cognitive impairment in aging [13], and in AD [25,26,27,28]. We reported earlier that ACD in older humans is associated with GSH deficiency, and that supplementing GlyNAC reversed GSH deficiency and improved cognition [13]. This study finds that the brains of old mice had decreased protein expression of the enzymes regulating GSH synthesis, and this was associated with severe GSH deficiency in the brain. Supplementing GlyNAC in old mice successfully improved the protein expression of enzymes responsible for brain GSH synthesis, and corrected GSH deficiency in the brain.

#### 4.3.2. Oxidative Stress (OxS)

OxS is a harmful state which rises from the excess accumulation of ROS generated by mitochondria during the process of energy generation. The initially formed ROS is the superoxide radical which is immediately detoxified by superoxide dismutase to hydrogen peroxide (H_2_O_2_). However, hydrogen peroxide is still a highly toxic component of ROS. GSH plays a critical role in cellular protection from the harmful OxS by neutralizing toxic hydrogen peroxide to water (as shown in the reaction H_2_O_2_ + 2GSH → 2H_2_O + GSSG). However, the term ROS per se is not synonymous with toxicity because ROS also performs beneficial functions by participating in cell signaling. The cell maintains a small concentration of ROS for this purpose, and an excess reduction of cellular ROS can also result in harm, known as ‘Reductive stress’ (RedS). This study found that GlyNAC supplementation successfully improved brain OxS (by lowering the increased TBARS concentrations in old mice) but did not induce RedS (because TBARS concentrations in old mice did not decrease below levels seen in young mice). By successfully lowering OxS and simultaneously preventing RedS, GlyNAC could be the ideal supplement to maintain and support cellular redox balance in aging.

### 4.4. GlyNAC Supplementation Reverses Abnormalities in Brain Glucose Metabolism

Although the brain represents about 2% of the body weight, it receives 25% of the blood supply suggesting that the brain requires a very high amount of energy. It has been reported that a single neuron in the resting brain requires about 4.7 billion ATPs per second [75]. Evidence from recent research suggests that this estimate could be incorrect by a factor of a billion [76]. Collective these data attest to an extremely high energy requirement by the brain. Glucose is the primary fuel substrate for brain energy generation. The brain critically depends on an uninterrupted supply of glucose as the primary fuel substrate to generate energy, and even small perturbations in glucose availability or mitochondrial function could severely limit the availability of energy needed for supporting brain and cognitive function. Our study in old mice investigated abnormalities in brain glucose metabolism in terms of (a) brain glucose supply (i.e., brain glucose transporter availability), and (b) brain mitochondrial function and energetics.

Glucose enters the brain by crossing the blood-brain barrier and then enters the neurons and brain cells where it is a fuel substrate for mitochondrial energy generation. The family of glucose transporters (GLUT) regulates glucose entry into cells. GLUT1 is expressed in the blood-brain barrier and in brain astrocytes, and GLUT3 is expressed in brain neurons. GLUT1 expression in the brain is reported to decline with aging [57,58,59,60] and in AD [61,62,63]. This study found evidence of lower protein expression of GLUT1 and GLUT3 in the brains of old mice and this indicates a critical barrier for glucose entry into the brain, astrocytes and neurons. Deficient glucose availability is likely to result in devastating consequences for the brain by limiting energy generation in mitochondria, and thereby adversely affecting neuronal function and brain health. Therefore, the discovery that GlyNAC supplementation in old mice significantly improved both brain GLUT1 and GLUT3 protein expression becomes relevant as it implies restoration of brain glucose availability. These findings are likely to have important implications for reversing similar defects in the brains of older humans, and potentially in patients with AD, because decreased expression of glucose transporters are reported in both conditions [57,58,59,60,61,62,63]. After entering the brain, glucose has to be oxidized by brain mitochondria to generate energy, and this is discussed next.

### 4.5. GlyNAC Supplementation Corrects Mitochondrial Dysfunction in the Aging Brain

Mitochondria are cellular engines that generate energy that is needed for sustaining optimal cellular function. ACD and AD are both reported to have mitochondrial dysfunction [13,77], suggesting that limited energy availability could be a key contributor to cognitive impairment and implying that reversing mitochondrial dysfunction in the brain could improve cognition. Age-associated mitochondrial impairment is associated with other defects linked to impaired cognition, such as elevated inflammation [78,79,80] and glucose dysmetabolism [22]. Optimal mitochondrial function can be envisioned as three processes occurring simultaneously: (a) generation of new mitochondria by a process of mitochondrial biogenesis; (b) efficient function of existing mitochondria to generate energy; (c) removal of nonfunctional mitochondria by a process known as mitophagy. This study found that the brains of old mice had abnormalities in all three aspects of mitochondrial function, and that GlyNAC supplementation reversed these abnormalities.

#### 4.5.1. Mitochondrial Biogenesis 

PGC1α is well established as a master regulator of mitochondrial biogenesis, mitochondrial quality and energy metabolism [81,82,83]. This study found low protein expression of PGC1α in the brains of aged mice suggesting diminished mitochondrial biogenesis and mitochondrial dysfunction. Brain PGC1α protein expression was increased and restored after GlyNAC supplementation, suggesting improvements in mitochondrial biogenesis and function.

#### 4.5.2. Mitochondrial Function 

Compared to young mice, the mitochondria in the brains of old mice had diminished protein expression of complexes I, II and V. Mitochondrial respirometry studies showed a significant decrease in the ability of the aging brain to oxidize fuel substrates such as pyruvate, glycerol phosphate, glutamate and succinate as the functional consequences of the decreased availability of these mitochondrial complexes. Although immunoblot analysis did not show a decrease in the protein expression of mitochondrial complex III, respirometry studies using inhibitors (antimycin-A and sodium azide) and stimulators (ascorbate-TMPD) indicate a functional defect in complex III. Therefore, this study found defective functioning of mitochondrial complexes I, II, III and V indicating that the brains of old mice had severe mitochondrial dysfunction. This would inevitably result in lower ability to generate energy, as suggested by decreased expression of ATP5A. GlyNAC supplementation significantly improved protein expression of complexes I, II, V and ATP5A, and the functioning of complexes I, II, III and V as assessed by mitochondrial respirometry studies. This study provides exciting new evidence that GlyNAC supplementation is able to reverse mitochondrial dysfunction in the aging brain, and this finding has important implications for improving cognition and supporting brain health in aging.

#### 4.5.3. Mitophagy

The PTEN-induced kinase 1 (PINK1) is a mitochondrial-targeted kinase that recruits the E3 ubiquitin ligase Parkin to mitochondria to initiate mitophagy [84,85]. This study found decreased protein expression of PINK1 suggesting decreased mitophagy in the aging brain. GlyNAC supplementation led to the recovery of PINK1 suggesting improvement in brain mitophagy. Improving mitophagy in the brain would result in optimizing and improving overall brain mitochondrial function, and thereby improve brain health and cognitive function.

#### 4.5.4. Implications of the Ability of GlyNAC Supplementation to Correct Mitochondrial Dysfunction in the Aging Brain

Collectively, these data indicate the presence of multiple defects in the regulation of mitochondrial biogenesis, availability and functionality of multiple mitochondrial complexes, and clearance of dysfunctional mitochondrial in the brains of old mice. These defects indicate severe mitochondrial function at multiple levels in the brain. This could limit brain processes that critically depend on energy availability and contribute to cognitive impairment. GlyNAC supplementation improves/corrects these defects to reverse mitochondrial dysfunction in the aging brain, and thereby supports brain health. These findings have the potential for immediate translation to humans because we recently reported in a randomized clinical trial that similar mitochondrial defects occur in the skeletal muscle of older adults and are corrected after GlyNAC supplementation [64]. We also reported earlier in a small pilot trial that GlyNAC supplementation can improve mitochondrial function and cognitive decline in older humans [13]. Accumulating evidence from aged mice and older humans provides proof-of-concept that GlyNAC supplementation can promote, support and improve brain health and cognition in aging. 

### 4.6. GlyNAC Supplementation Improves Abnormal Brain Autophagy and Mitophagy

Autophagy is a cellular mechanism of removing cellular debris in the form of dysfunctional organelles such as mitochondria (mitophagy), and removal of other accumulated non-functional cellular components. Autophagy is mediated by lysosomes which engulf and degrade cellular debris, and this process involves multiple discrete steps. One of the molecular intermediates is known as LC3A/B (LC3-I/II), and is a validated marker for autophagy [86,87]. Our study found that LC3A/B protein expression was diminished in the brains of aged mice suggesting impaired autophagy. There was also a concomitant decrease in the protein expression of PINK1, suggesting impaired mitophagy. Supplementation with GlyNAC led to improved protein expressions of both LC3A/B and PINK1 suggesting improvement/restoration of autophagy and mitophagy in the aging brain. The overall implications of these findings are an improved ability of the brain to remove cellular debris, and thereby promote cellular function and brain health.

### 4.7. GlyNAC Supplementation Lowers Inflammation in the Aging Brain

Chronic, elevated inflammation is associated with aging [11,13,20,21,23,24]. The translocator protein (TSPO) is widely used as a marker for neuroinflammation [88,89,90]. Elevated inflammation is well recognized as a key defect in aging, and this study found that the brains of aged mice had severely elevated inflammation (as increased TSPO protein expression). One hypothesis for the increased brain inflammation is that it is mediated by the activation of brain microglia. Iba1 is a pan-microglial marker whose expression increases with microglial activation [91,92]. To understand whether microglial activation was responsible for increased inflammation in the aging brain, we measured the protein expression of microglial Iba1 and found no significant differences between the brains of young and old mice and no response to GlyNAC, suggesting that microglial activation is not the prime driver of inflammation in the aging brain. Inflammation per se is linked to mitochondrial dysfunction which also promotes OxS, and in turn OxS is reported to further increase inflammation. At the center of these defects lies glutathione deficiency, and we previously reported in human clinical trials that correcting GSH deficiency with GlyNAC also improves mitochondrial dysfunction, lowers OxS and improves multiple inflammatory cytokines [13,64]. Similar observations were seen in this study where lowering of brain inflammation after GlyNAC supplementation occurred in parallel with correction of brain GSH deficiency, lowering of OxS and restoration of mitochondrial function. Elevated inflammation in the context of aging (termed ‘inflammaging’) is linked to multiple defects in organ function. The association of cognitive decline with elevated brain inflammation suggests a contributory role for inflammation for cognitive decline in aging. Evidence to support causality between the improvement of inflammation and GlyNAC supplementation comes from our human trials which showed that GlyNAC supplementation in older adults significantly lowered multiple circulating pro-inflammatory cytokines [13,64], but stopping GlyNAC supplementation led to a rise in inflammation [13].

### 4.8. GlyNAC Supplementation Lowers Genomic Damage in the Aging Brain

Mammalian DNA can be damaged by OxS [93,94], and the ensuing genomic damage is believed to contribute to the aging process and is identified as an ‘aging hallmark’ [20,21]. Damage to the genome can have a widespread impact by inducing defects in cellular processes and organ function, which are common in aging. In this study, we measured genomic damage in the brain as protein expression of pH2AX and found evidence of increased genomic damage in the aging brain. GlyNAC supplementation in old mice lowered the protein expression of brain pH2AX suggesting a reduction in brain genomic damage, and thereby, an improvement in brain health. The study also found that lower brain genomic damage was associated with improved cognitive function in aged mice suggesting that genomic damage could be a contributor to cognitive decline in aging.

### 4.9. GlyNAC Supplementation Improves Neurotrophic Factors in the Aging Brain

Neurotrophic factors in the brain are believed to play an essential role in supporting neuronal survival and proliferation, dendritic and axonal growth and patterning, synaptic strength and plasticity, and function by signaling via tyrosine kinase receptors [95,96,97,98]. In this study, we evaluated three neurotrophic factors, brain-derived neurotrophic factor (BDNF), glial-derived neurotrophic factor (GDNF) and nerve growth factor (NGF). The protein expression of BDNF, GDNF and NGF were all significantly lower in the aging brain, and all were rescued by GlyNAC supplementation. This discovery could have significant implications for improving brain health in aging due to the functional role played by these neurotrophic factors in promoting and maintaining brain health. 

BDNF has been extensively studied in neuroscience and is believed to play a key role in the formation of new neurons and maintaining the health of existing neurons, neuronal development, synaptogenesis, neuroprotection, memory and cognition [99,100,101]. We reported earlier that lower cognitive test scores in older humans were associated with lower circulating BDNF levels, and that GlyNAC supplementation improved BDNF levels and cognitive performance, but benefits receded on stopping GlyNAC, indicating causality [13]. A more recent study found that BDNF mediates improvement in cognitive performance after computerized cognitive training in healthy older adults [102]. BDNF is also reported to promote the formation of new neurons [103], which may offer exciting new hope in terms of replacing damaged, lost or nonfunctional neurons. ‘BDNF-mimetic’ development is being considered to reverse BDNF deficiency in the brain to improve brain health, memory and cognition [104]. It is from this context that the results of our study assume significance, because GlyNAC supplementation improved natural endogenous BDNF levels to correct its deficiency in the aging brain, and this was associated with improvements in cognition and other brain defects. Therefore, GlyNAC supplementation could play a key role in promoting brain health and cognition in aging. 

GDNF is identified as being important for preserving the health of dopaminergic neurons, with implications for Parkinson’s disease [105,106,107,108,109], but there appears to be limited clinical success of exogenously administered GDNF [110,111]. Another interesting study reported that ischemia-injured neurons were rescued by astrocytic GDNF modulation [112] suggesting a wider role for GDNF in preserving and promoting brain health and recovery. Therefore, it is interesting to find that the aged mice in this study had low expression of GDNF in the brain and that GDNF expression improved to levels seen in young mice after GlyNAC supplementation. This finding indicates restoration of endogenous GDNF in their native environment rather than provision via exogenous administration. While it may be premature to consider the implications of GlyNAC supplementation in Parkinson’s disease based on this study which did not measure GDNF specifically in dopaminergic neurons, the findings of this study nevertheless support the need for future research on the effect of GlyNAC supplementation in dopaminergic neurons and signaling pathways. 

NGF dysfunction is reported in aging, MCI and AD [113,114,115], and may play a key role in neurodegeneration. 

Improving or reversing neurotrophic factor deficiency is being considered as potentially viable options for a wide range of neurological conditions including brain injury, AD and Parkinson’s disease [116,117,118,119,120,121]. The results of this study provide exciting new knowledge that GlyNAC supplementation can successfully improve and reverse the deficient protein expression of BDNF, GDNF and NGF in the aging brain and thereby play a key role in maintaining and promoting brain health in aging.

### 4.10. Why GlyNAC Works—The ‘Power of 3’

GlyNAC is a combination of glycine and cysteine (provided as NAC, a cysteine donor) as precursors necessary for glutathione synthesis. GSH deficiency cannot be corrected by oral ingestion because it is digested in the gut. Because cells have to maintain a delicate balance between avoiding oxidative stress (due to elevated levels of ROS) and avoiding reductive stress (due to excess decrease in ROS levels) they have to necessarily maintain the ability to regulate their own GSH synthesis and concentrations. This is the likely reason why every cell in every organ makes its own GSH and does not depend on delivery via blood, or exogenous administration of GSH. Excess administration of GSH alone and NAC alone has been shown to accelerate aging in *C. elegans* [122], while we reported earlier that GlyNAC supplementation in our rodent studies increases natural production of intracellular GSH and extends life [14]. GlyNAC supplementation also restores GSH adequacy in multiple tissues such as the heart, liver, kidney, skeletal muscle, and red-blood cells to lower OxS without inducing reductive stress [11,12,13,14,64]. Similar findings were seen in the aging brain in this study where GlyNAC supplementation corrected impaired expression of enzymes regulating GSH synthesis to correct GSH deficiency and lower OxS. It is important to note that brain GSH levels in GlyNAC-supplemented aged mice did not increase above that in young mice indicating that the cellular autoregulation to maintain optimal levels of intracellular GSH was intact. Similarly, ROS levels (as TBARS concentrations) in the brains of aged mice also did not decline below young mice after GlyNAC supplementation, suggesting avoidance of reductive stress. Because GlyNAC depends on cells to make their own GSH and thereby corrected GSH deficiency naturally, it could be the ideal supplement for GSH maintenance in aging brains.

Glycine is an important amino acid and is reported to be rate-limiting for GSH synthesis in aging [12,123]. Glycine plays an important role in supporting health [124], functions as a neurotransmitter in the brain, and also supports synaptic function [125,126,127,128]. Cysteine is an important amino acid that is deficient in aging [12,129]. Cysteine functions by donating the sulfhydryl (SH) group and plays a key role in many thiol reactions, especially in mitochondria [130,131,132,133,134]. GSH is the most abundant endogenous intracellular antioxidant, plays a key role in cellular protection, and confers other health benefits [135,136]. The combination of glycine, cysteine and GSH, is referred to as the ‘*Power of 3*’ and is provided by GlyNAC supplementation. We previously reported multiple benefits of GlyNAC supplementation in rodent studies and human clinical trials in aging [11,12,13,14,22,64]. This study provides additional evidence on the benefit of GlyNAC supplementation in aging in terms of improving cognitive function and promoting brain health.

### 4.11. A Proposed Model for the Development of Cognitive Decline

**Figure 14:**
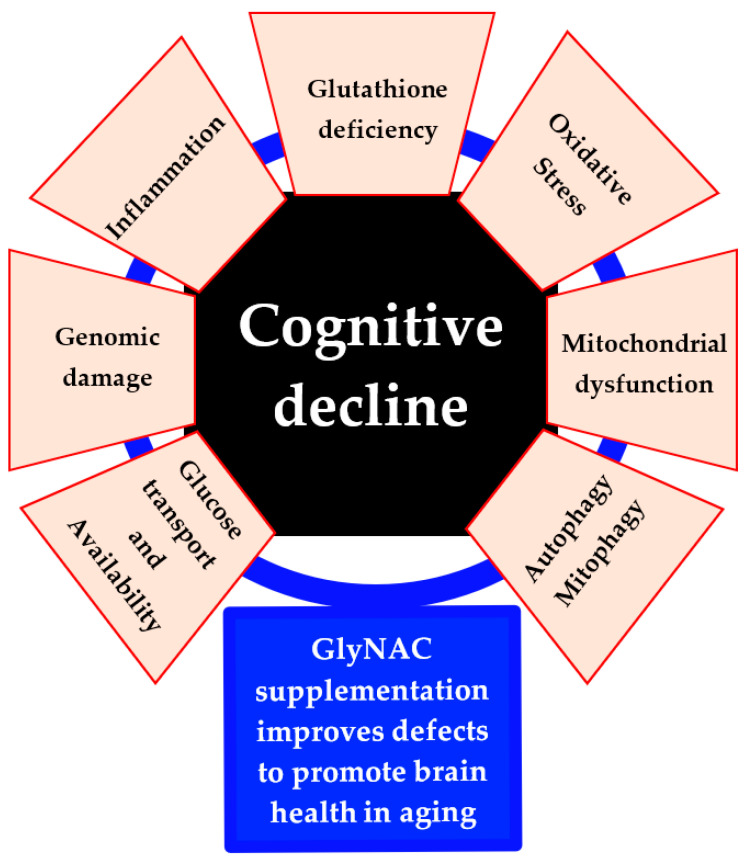
A working model for the mechanistic origins of cognitive decline, and reversibility after supplementation with GlyNAC. The blue ring denotes potential interactions between these defects which result in cognitive decline.

Evidence from the current study and our earlier small pilot trials in older adults and cognitively-impaired HIV patients [13,137] indicate that cognitive decline is associated with several specific defects involving glutathione deficiency, elevated oxidative stress, mitochondrial dysfunction, abnormal autophagy and mitophagy, impaired brain glucose availability and uptake, and elevations in inflammation, endothelial dysfunction and genomic damage. Similar defects are also described in AD [25,26,27,28,29,30,31,32,33,34,35,36,37,38,39,40,41]. These findings suggest that a combination of defects could be responsible for cognitive decline. We hypothesize that there is a critical contribution stemming from each defect which combines to initiate cognitive decline (Figure 14). By improving/reversing each discrete defect, GlyNAC supplementation also improves/reverses cognitive decline. This proposed model is supported by data from this rodent study, and from our prior trial in older humans where improvements in these defects and cognitive function after GlyNAC supplementation were reversed after GlyNAC withdrawal, suggesting causality [13]. Future clinical trials are needed to determine whether GlyNAC supplementation in older adults could improve the defects outlined in this model and thereby improve/reverse cognitive decline in aging, and also potentially in AD.

### 4.12. Implications for Alzheimer’s Disease and Mild Cognitive Impairment (MCI)

The findings of this study and the proposed model could have implications for AD and MCI because of the following: (a) defects occurred in aging brains, and this is relevant because increased age is identified as the most important risk factor for AD [1,2,3,4]; (b) similar defects are reported in AD [25,26,27,28,29,30,31,32,33,34,35,36,37,38,39,40,41]; (c) imaging studies confirm the overlap of glucose hypometabolism and accumulation of the tau protein in the brains of patients with AD; (d) GlyNAC supplementation improved brain defects in aged mice and also improved cognition; similar improvements were reported in a small pilot study in aging humans with ACD [13]. Based on these data we propose two hypotheses for AD: (1) GlyNAC supplementation could improve GSH deficiency, OxS, mitochondrial dysfunction, inflammation, endothelial dysfunction, and brain glucose uptake as mechanistic defects associated with and contributing to impaired cognition; (2) GlyNAC supplementation to reverse ACD could prevent the development of AD. These hypotheses warrant testing in future clinical trials.

### 4.13. Study Limitations

Although the observations in this rodent study provide evidence that supplementing GlyNAC improves age-associated cognitive decline and underlying contributory defects in the brain, the study was limited to male mice. However, our previous trials showed that GlyNAC supplementation improved cognition and additional age-associated defects in both men and women [13,64]. Future rodent studies evaluating the effects of GlyNAC supplementation on brain health and cognition should include both genders.

## 5. Conclusions

The results of this study provide proof-of-concept that GlyNAC supplementation improves age-associated cognitive decline and supports brain health in aging by improving/correcting brain GSH deficiency, elevated OxS, mitochondrial dysfunction, abnormal mitophagy and autophagy, inflammation, impaired glucose transport/uptake, elevated genomic damage, and reversing the decline in brain neurotrophic growth factors. GlyNAC could be a simple and effective nutritional supplement to support brain health and cognitive function in aging, and warrants additional investigation.

## Figures and Tables

**Table 1:** Glutathione concentrations in the brains of young mice (Y), old-control mice (OC) and old mice supplemented with GlyNAC (OG). t-GSH, r-GSH and GSSG are the total, reduced and oxidized glutathione concentrations. Results are reported as mean ± SE (standard error) and considered to be statistically significant at values *p* < 0.05.

	Y *YC* vs. *OG*	OC *YC* vs. *OC*	OG *OC* vs. *OG*
t-GSH (μmol/g)	6.4 ± 0.8 *p* = 0.6	2.9 ± 0.5 *p* < 0.01	6.0 ± 0.8 *p* < 0.01
r-GSH (μmol/g)	4.3 ± 0.2 *p* = 0.6	1.9 ± 0.1 *p* < 0.001	4.1 ± 0.4 *p* < 0.001
GSSG (μmol/g)	2.1 ± 0.7 *p* = 0.77	1.0 ± 0.3 *p* = 0.42	1.9 ± 0.6 *p* = 0.47

## Data Availability

All relevant data are contained in this manuscript.
